# 

**Published:** 1901-01

**Authors:** 


### ILLUSTRATIONS.

| | PAGE |
|---|---|
| A. V. M. A., Annual Meeting . . . . . . . . . . | 441 |
| Canine Instruments, Some New . . . . . . . . | 162, 163, 164 |
| Direct Compound Comminuted Fracture, with Indirect Simple Fracture of the Inferior Maxilla . . . . . . . . . . | 45 |
| Editorials . . . . . . . . . . . . . . | 519, 524, 525 |
| Extra-uterine Fœtation in the Bitch . . . . . . . . | 727 |
| Fleming, George . . . . . . . . . . . . . | 273 |
| National Disgrace, A . . . . . . . . . . . . | 519 |
| Recurrence of Estruation in the Bitch after Oöphorectomy, On the . | 285 |
| Thyroid Gland and Thyroid Glandules of the Dog . . . . | 4, 5 |

### EDITORIAL.

### MARRIAGES.

Mr. Alexander Eger, the well-known veterinary book publisher, of Chicago, Ill., was married to Miss Vilma Glick, of the same city, on Wednesday, January 2, 1901.

At Newark, N. J., January 2d, Dr. James T. Glennon to Miss Mary E. Quinn. Dr. Glennon is a graduate of the New York College of Veterinary Surgeons, class of 1896, and at present veterinarian to the Newark City Fire Department.

Secretary.

### PERSONALS.

Dr. R. S. Huidekoper was confined to his bed for two weeks in December from an acute attack of pneumonia.

Dr. E. A. A. Grange has a leading article in the January *Cosmopolitan* on "How to Judge a Horse."

Dr. William Roehrig, of Philadelphia, has been seriously ill during the past three months, and was confined to one of the leading hospitals for some weeks.

Dr. H. E. Rowell was the purchaser of "Wallenstein," one of the noted running horses of the late Marcus Daly.

Dr. Leonard Pearson, State Veterinarian of Pennsylvania, spent Christmas at the National Capital.

Dr. L. L. Conkey, dean of the Grand Rapids Veterinary College, was once an applicant for admission as a student to the Ontario Veterinary College.

Dr. James A. Waugh, of Pittsburg, is rejoicing over the advent of another daughter. He reports practice as very good indeed. Blessings never go singlehanded in the pathway of our friend Waugh.

Dr. and Mrs. W. Howard Wilson, of Hatboro, Pa., have just returned from their wedding trip.

Dr. Walter L. Hart, city veterinarian, was confined to one of the Philadelphia hospitals during the early part of December from an affection of the heart.

Dr. Cecil French, of Washington, D. C., spent the Christmas holiday season in Montreal, Canada, including in his trip a visit to Toronto, Niagara Falls, and Buffalo, where the progress of the Exposition was inspected.

Dr. D. S. White, of Columbus, Ohio, will complete the translation of "Outline of the Clinical Diagnosis of the Internal Diseases of Domestic Animals," commenced by Professor Olof Schwarzkopf, whose army service has prevented its completion.

Dr. Charles H. Higgins resumed his special laboratory work in relation to bubonic plague at Montreal, Canada, early in December.

Dr. E. M. Ranck, of Philadelphia, takes an active interest in municipal politics, and has become the secretary of a recently formed political organization.

Drs. F. F. Hoffman, of Brookville, Pa.; Jacob Helmer, of Scranton, and J. W. Sallade, of Pottsville, were visitors to Philadelphia in December.

Dr. T. W. Spranklin, of Baltimore, Md., was a visitor to Philadelphia early in December. A son at the Veterinary Department of the University of Pennsylvania leads to frequent visits to the "Quaker City."

Dr. G. R. Hartman, of Philadelphia, was among the exhibitors of St. Bernards at the Philadelphia dog show in November.

Dr. R. W. Hickman, formerly of New York, has been transferred to Washington, D. C., to become chief of the Miscellaneous Division of the Bureau of Animal Industry.

Dr. Otto G. Noack, of Reading, Pa., was a visitor to the fat-cattle show at Philadelphia in the latter part of November.

Dr. Leonard Pearson, State Veterinarian, was a visitor to Harrisburg early in December.

